# Relationship between Cocoa Intake and Healthy Status: A Pilot Study in University Students

**DOI:** 10.3390/molecules24040812

**Published:** 2019-02-23

**Authors:** Maria J. Rodríguez-Lagunas, Filipa Vicente, Paula Pereira, Margarida Castell, Francisco J. Pérez-Cano

**Affiliations:** 1 Departament de Bioquímica i Fisiologia, Facultat de Farmàcia i Ciències de l’Alimentació, Universitat de Barcelona, 08028 Barcelona, Spain; mjrodriguez@ub.edu (M.J.R.-L.); franciscoperez@ub.edu (F.J.P.-C.); 2 Institut de Recerca en Nutrició i Seguretat Alimentària, Universitat de Barcelona (INSA-UB), 08921 Santa Coloma de Gramenet, Spain; 3 Centro de Investigação Interdisciplinar Egas Moniz, Egas Moniz Cooperativa de Ensino Superior, Quinta da Granja—Campus Universitário, 2829-511 Monte da Caparica, Portugal; fvicente@egasmoniz.edu.pt (F.V.); pmpereira@egasmoniz.edu.pt (P.P.)

**Keywords:** allergy, cacao, chocolate, food frequency questionnaire, International Physical Activity Questionnaire, young people

## Abstract

Due to its polyphenol content, cocoa’s potential health effects are attracting much attention, showing, among other things, cardioprotective, anti-inflammatory, anti-obesity, and neuroprotective actions. However, there is very limited information regarding the effect of cocoa on human immunity. This study aimed to establish the relationship between cocoa consumption and health status, focusing on physical activity habits and allergy prevalence in young people. For this, a sample of 270 university students was recruited to complete a food frequency questionnaire, the International Physical Activity Questionnaire (IPAQ), and a lifestyle and health status questionnaire. The results were analysed by classifying the participants into tertiles defined according to their cocoa consumption: low (LC), moderate (MC), and high (HC) consumers. The consumption of cocoa inversely correlated with physical activity and the MC group had significantly less chronic disease frequency than the LC group. The percentage of allergic people in the MC and HC groups was lower than that in the LC group and, moreover, the cocoa intake, especially moderate consumption, was also associated with a lower presence of allergic symptoms. Thus, from these results a positive effect of cocoa intake on allergy can be suggested in the young population.

## 1. Introduction

Polyphenols are naturally-occurring compounds in vegetal food products such as fruits, vegetables and cereals, and also in specific beverages such as wine, tea, coffee and cocoa [1,2,3]. Over recent years, the relationship between polyphenol-rich food consumption and human health has been closely established [4]. This fact has led to the consideration that a healthy diet, such as a Mediterranean diet, must contain a high intake of phenolic compounds, which are present in fruits, vegetables, wholegrain cereals, nuts, olive oil and also in red wine, coffee and tea [5]. Indeed, the growing interest in the role of polyphenols in a healthy human diet has given rise to the discovery of novel foods enriched with polyphenols extracted from different natural sources, both raw materials and by-products coming from several food chains [6], such as from olives and citrus fruits [7,8].

Cocoa is one of the most polyphenol-rich foods [9,10] and some data indicate that polyphenols constitute 12–18% of the whole cocoa bean’s dry weight [11]. Polyphenols in cocoa beans belong to the flavonoid family and mainly include flavan-3-ols, presented as monomers, such as (+)− and (−)− isomers of epicatechin and catechin, and polymeric forms which are a build-up of epicatechin subunits [9,12]. Minor cocoa components are phenolic acids, flavonols, and stilbenes, among others [12].

Chocolate derives from the fermentation, roasting, and milling of cocoa beans [13]. In the next stage, a mixing process combines the milled cocoa liquor with sugar. Depending on the final chocolate product, ranging from white to dark chocolate, the quantities of ingredients vary significantly. Whereas white chocolate is only cocoa butter, milk chocolate contains a significant quantity of milk, and dark chocolate contains cocoa solids in quantities from as low as 40% to as high as 100% [13]. Consequently, the polyphenol content in the chocolate will increase in direct proportion to the cocoa powder content. According to the Phenol Explorer database [14], cocoa powder has a flavanol content of about 510 mg/100 g, with (-)-epicatechin (158.30 mg/100 g), procyanidin B1 (112.00 mg/100 g), (+)-catechin (107.75 mg/100 g), procyanidin B2 (71.57 mg/100 g) being the most important. In the case of dark chocolate, the main phenolic composition (about 240 mg/100 g) depends on (-)-epicatechin (70.36 mg/100 g), cinnamtannin A2 (53.83 mg/100 g), procyanidin B2 (36.50 mg/100 g), procyanidin C1 (26.00 mg/100 g), quercetin (25.00 mg/100 g), and (+)-catechin (20.50 mg/100 g) [14]. In the case of white chocolate, the content of polyphenols established (about 20 mg/100 g) is limited to (-)-epicatechin (14.58 mg/100 g) and (+)-catechin (4.64 mg/100 g) [14].

In recent years, due to its polyphenol content, cocoa has gained increased attention regarding its potential health effects [12]. Of the beneficial effects of cocoa, the antioxidant effect derived from its polyphenols is one of the best studied properties [15]. It has been estimated that a serving of dark chocolate (40 g) provides the antioxidant capacity equivalent to 9100 Trolox [16] and can provide more phenolic antioxidants than beverages and fruits such as tea and blueberries, traditionally considered high in antioxidants [9,17]. In addition, cocoa and dark chocolate consumption has shown to be associated with cardioprotective, anti-inflammatory, anti-obesity, anti-carcinogenic and neuroprotective actions in addition to a potential role in carbohydrate and lipoprotein metabolism regulation [18,19,20,21,22,23]. These associations have mainly been performed in populations with disease risk or in patients.

There is limited information regarding the effects of cocoa on human immunity, but preclinical studies have demonstrated that cocoa possesses immunoregulatory properties both at systemic and intestinal levels [24,25]. Among these effects, it has been demonstrated that a cocoa-enriched diet can down-regulate allergy-related antibody synthesis [25,26]. These immunoprotective actions of cocoa have not yet been demonstrated in humans, either by observational or interventional studies.

To increase current knowledge about the relationship between cocoa intake and health and to shed light on the immunoprotective actions of this food, a cross-sectional, observational, pilot study was carried out. This study was focused on establishing the relationship between amount of cocoa consumption and health status, focusing on physical activity habits and allergy prevalence in young people. As far as we know, this is the first time that such an observational study to establish the relationship between cocoa intake and its possible immunoprotective actions has been carried out. For this, a total of 270 students from two universities in Spain and Portugal were required to respond to a previously described food frequency questionnaire (FFQ) aimed at establishing cocoa consumption habits [27], the International Physical Activity Questionnaire (IPAQ), and a lifestyle and health status questionnaire. Then the results were analysed by classifying the participants into tertiles defined according to their cocoa consumption.

## 2. Results

### 2.1. Population Distribution according to Cocoa Consumption

The study population was young people recruited from several undergraduate and postgraduate programmes at the University of Barcelona and in the Egas Moniz Health Sciences Institute of Portugal. The 270 students (199 females and 71 males, 22.5 ± 3.9 years old on average—mean ± standard deviation of the mean, SDM) were ordered according to cocoa consumption, and distributed into three groups: ‘Low consumers’ (LC), ‘Moderate consumers’ (MC) and ‘High consumers’ (HC). The LC group consumed less than 7 g of cocoa/day, the MC group consumed 7–15 g of cocoa/day and finally, the HC group consumed more than 15 g of cocoa/day (Table 1). Overall, the mean consumption of this particular population was about 13 g/day. There were no differences regarding lifestyle of participants among the groups, such as residential status (mainly living with family, >50% in all cases) and being less than 18% occupied in all groups.

### 2.2. Cocoa Consumption and Body Weight and Smoking Habits

**Figure 1:**
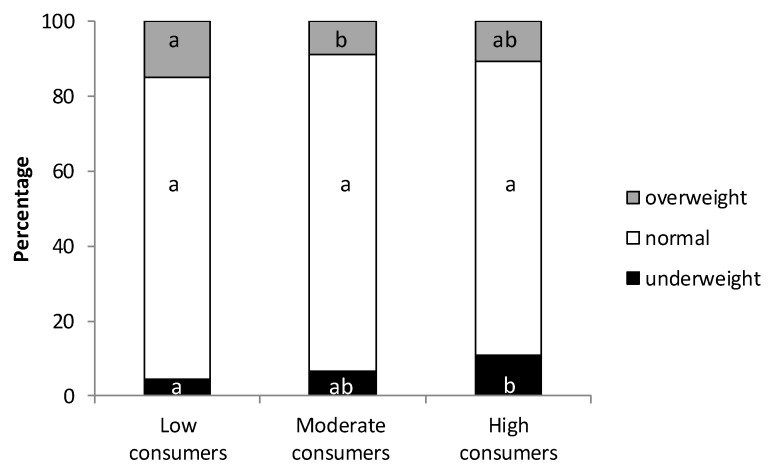
Percentage of normal, overweight and underweight students divided into three groups according to their cocoa consumption. Different letters mean statistical difference.

The mean body mass index (BMI) was calculated for each group of cocoa consumers and no significant differences between them were found (Table 2). However, when the number of underweight (BMI < 18.5), normal (18.5 ≤ BMI ≤ 25) and overweight (BMI > 25) participants were assessed, a significantly higher proportion of underweight students was found in the HC group in comparison to the LC group (Figure 1) (*p* < 0.05). In addition, the lowest proportion of overweight individuals was found in the MC group (*p* < 0.05 with respect to LC group). Regardless of these effects, and although a weak inverse tendency correlating cocoa consumption and BMI was found, no statistical association by Spearman’s test was reached. Nevertheless, these results show that high cocoa consumption was not accompanied by higher BMI in the population studied.

Students were also asked about their smoking habits and subsequently categorized into the following groups: non-smoker, smoker and ex-smoker (Table 2). About 20% of university students declared they were smokers. No differences appeared between cocoa consumer groups when considering the non-smokers and the current smokers. However, interestingly, the proportion of ex-smokers was higher in the MC and HC groups than that in the LC students.

### 2.3. Cocoa Consumption and Physical Activity

Physical activity usually performed by students was evaluated using the IPAQ short version. This questionnaire allows physical activity to be measured in median minutes or median metabolic equivalents of task (METs). The mean physical activity of all participants was 2692 ± 2549 METs/week. After categorizing the students according to three physical activity levels, the whole population was distributed as follows: 18% as low active, 39% as moderate active, and 42% as highly active. Then the METs/week and the categorical activity classification (low, moderate and high activity) in each cocoa consumption group (LC, MC and HC) were studied.

No differences were observed in the total METs spent per week among the groups (Table 2). However, and taking into account the cocoa consumption and METs of all participants, a tendency for an inverse correlation was found (*p* = 0.087, Spearman’s test), thus, the more cocoa participants ate, the less exercise they performed.

**Figure 2:**
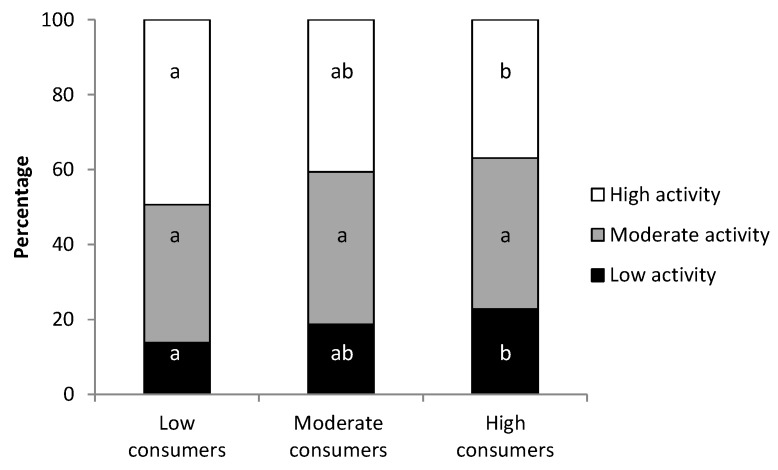
Percentage of students with low, moderate and high activity according to their cocoa consumption. Different letters mean statistical difference.

In line with this, when considering the physical activity categories (Figure 2), the LC group had the highest proportion of active students (~50%) and the lowest proportion of inactive students (~14%), whereas the HC group included the lowest proportion of highly active participants (~37%) and the highest proportion of students with low activity (~23%). The proportion of students with high and low activity differed significantly according to whether they belonged to the low or high consumer group (*p* < 0.05).

In the IPAQ, the students were also asked about their sitting time throughout the day. This value was about 6–7 h/day and was similar among the three groups analysed.

### 2.4. Cocoa Consumption and Disease

Participants were asked to answer a survey about their health status. As it has been reported that cocoa intake decreased blood pressure (BP) [28], students were asked about their usual BP values. Mean BP in the LC, MC and HC groups did not differ significantly between groups (Table 3). Moreover, considering the percentage of students with hypertension in relation to their cocoa consumption, the percentages obtained were very low (Table 3), as would be expected in young people, and there were no differences between groups.

On the other hand, more than 50% of students said that they suffered from a chronic disease, with headaches and migraines being the most prevalent chronic conditions reported in this questionnaire. The highest proportion of declared chronic diseases was found in the LC subgroup when compared to those groups eating higher amounts of cocoa (Table 3). However, only the percentage of MC suffering chronic disease (51%) was statistically lower than that in the LC (64%) (*p* < 0.05). On the other hand, although without reaching statistical significance, the MC group had a trend that showed fewer cases of fever or other illnesses than the other groups. Specifically, the MC group showed values around 2% lower than the proportions in the LC and HC groups (Table 3). Finally, around 30% of students referred to having suffered flu in the assessed period. When this incidence was analysed in each cocoa consumption group, no differences were detected. Likewise, no statistical differences were found regarding diarrhoea or illness in general.

### 2.5. Cocoa Consumption and Allergy

**Figure 3:**
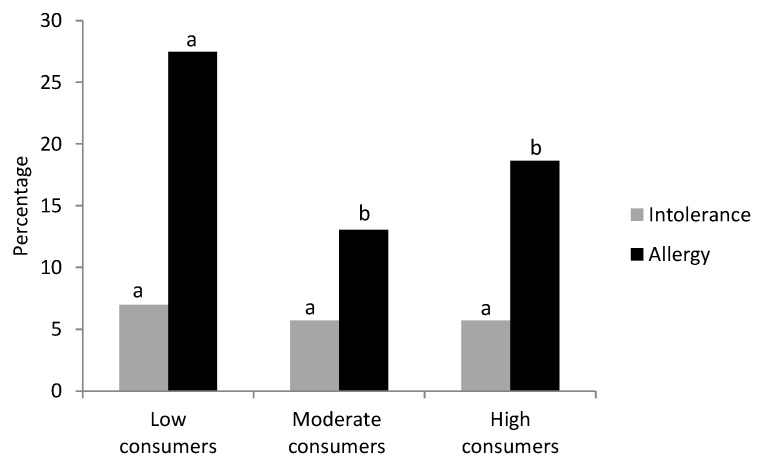
Percentage of students with food intolerance or allergy according to their cocoa consumption. Different letters mean statistical difference.

The questions included in the survey about health status also inquired about food intolerances, allergy and its symptoms. The proportion of students with food intolerance was about 5.5–7% and did not differ between LC, MC and HC groups (Figure 3).

Regarding allergies and taking into account all the participants’ information, approximately 20% declared they suffered some allergy. Interestingly, when allergy prevalence was calculated in each group according to cocoa consumption, statistical differences were found (Figure 3). Particularly, 24/87 students in the LC group reported allergy (28%) which was a higher proportion than that obtained in the MC and HC groups (13% and 19%, respectively, *p* < 0.05).

Considering the allergic people of each group, their allergy symptoms were analysed (Table 4). Firstly, the frequency (days per month) in which allergic students had symptoms was considered. Although the LC group had the highest frequency, the huge variability in each subgroup did not allow statistical differences in comparison with MC and HC groups to be demonstrated. Students were also asked about whether they have allergic symptoms at least once a month. Half or more of the allergic students of the LC and HC groups reported they suffered from allergy once a month, whereas this proportion was significantly lower in the allergic MC group (*p* < 0.05).

The survey about health status also inquired about the type of allergic symptoms suffered by the allergic students (Table 4). About 36% of all allergic students reported having developed cutaneous symptoms such as hives, redness or itching. Comparing students according to their cocoa consumption (Table 4), 42% of the allergic participants in the LC group had cutaneous symptoms, which was a significantly higher proportion than that in the MC allergic students (*p* < 0.05). In the HC group, the percentage of students with cutaneous symptoms lay between the middle of that in the LC and MC groups.

About 34% of students reported having developed rhinitis, sneezing or mouth itching, and comparing the proportion in each group of cocoa consumers, the LC group had higher proportion than the MC group (*p* < 0.05), whereas there were no differences with the HC group.

The number of allergic students with respiratory symptoms was about 11% and again the highest proportion was found in the LC group (*p* < 0.05 vs. MC group) although no differences were achieved with the HC group. No students reported having suffered from digestive symptoms due to allergy in any of the groups.

Finally, both the absenteeism derived from allergy episodes and whether the students were under allergy treatment in the last year were also inquired after. Of the 24 allergic subjects in the LC group, only three reported that they had to remain at home for 1–3 days due to the allergy. From the 12 allergic students in the MC group, none reported having been absent due to their allergy, and in the HC group, 2/15 students reported having stayed at home for 1 day. Thus, in line with previous results, the proportion of people with absenteeism caused by allergy was the lowest in the MC group, although no statistical differences were achieved.

Reinforcing the effects of a moderate consumption of cocoa on allergy, it is important to point out that more than half of the students consuming the lowest cocoa amounts and the highest cocoa amounts were under anti-allergy treatment. However, this percentage was only 33% in the moderate consumers (*p* < 0.05 vs. LC and HC groups).

## 3. Discussion

Cocoa consumption has an impact on health and, taking into account preclinical evidence [29,30,31,32], a potential preventive effect on immune-mediated diseases. In order to increase the current knowledge about the relationship between cocoa intake and health and to shed light on the immunoprotective actions of this food, a cross-sectional, observational, pilot study was carried out in young people. Two hundred and seventy students from two universities from two different countries answered a new validated FFQ to assess their amount of cocoa consumption [27], the IPAQ to quantify physical activity, and an ad hoc questionnaire to test health status. From the FFQ data, the amount of cocoa per portion within each FFQ item was calculated considering established portions or food labels in each food category, then the amount of cocoa per portion, and finally the amount of each one was converted into cocoa amount per portion [27]. Therefore, the estimated consumption obtained through the questionnaire was of about 13 g cocoa per day, which fits in with the great variability range of results found in the literature [27]. The amount of estimated cocoa consumption provides about 66 mg of flavanols per day [14]. The students were categorized into low consumers, consisting of those eating <7 g/day of cocoa (i.e., <36 mg of cocoa flavanols/day), moderate consumers, comprising those eating 7–15 g/day of cocoa (i.e., 36–76 mg of cocoa flavanols/day), and high consumers, those having an intake of >15 g/day of cocoa (i.e., >76 mg of cocoa flavanols/day). These cocoa amounts are equivalent to eating less than 5 g of 70%-cocoa chocolate, between 5 and 10 g of 70%-cocoa chocolate and more than 10 g of the same chocolate, respectively. However, it has to be taken into account that chocolate bars only provide 55% of cocoa in the diet, and other foods, such as dairy products, represent a source of up to 23% in this population [27].

Health status was assessed based on several criteria, including anthropometrical data. Excessive chocolate consumption is regularly seen as a hazard for weight control [33,34], although anti-obesity actions of cocoa have been reported [35,36,37]. We found that even though no significant changes in BMI were found among the three student groups, a lower proportion of overweight subjects appeared in the moderate consumer group and a higher proportion of underweight subjects appeared in the high consumer group. These results are in line with preclinical data obtained in rats from different strains, age and health conditions [38,39,40]. In these studies, after receiving a 10% cocoa diet with 4 g/100 g polyphenols (which could be considered ‘high consumer’ rats), a slower body weight increase has been consistently observed. Moreover, other studies in humans also reflect this behaviour. In this sense, in a young population (1458 adolescents aged from 12.5 to 17.5 years), the HELENA study demonstrated that higher chocolate consumption was associated with lower levels of BMI and lower total and central fatness [41].

The relationship between smoking and cocoa products’ consumption was also studied, and the results obtained were quite particular. Although no differences were found when considering the current smokers and students who had never smoked, the percentage of former smokers in moderate and high cocoa consumers was two to three times higher than that in low cocoa consumers, which may suggest that the role of cocoa as a sweet was being used to substitute the tobacco habit. In line with this, the former smoker may be looking for a sensory property that is lost after quitting tobacco, which can be found in cocoa because it is widely applied to cigarettes and has been used by the tobacco industry as an additive since the early twentieth century [42]. Interestingly, there is consistent evidence that stopping smoking is associated with increases in body weight and BMI [43], however this is not the case in this study, reinforcing the effect of cocoa consumption in the regulation of BMI.

From the results of the IPAQ, it can be observed that the range of physical activity in the university student populations was very wide. In this regard, it is important to highlight that there were students who reported very low physical activity, which would be hazardous to health promotion and disease prevention [44,45]. In fact, 14% of students had a sedentary lifestyle (<600 METs/week) and 40% did not reach the physical activity levels established by the recommendation guidelines for health promotion (1500 METs/week) [46]. However, these results are more favourable than those reported by Varela-Mato et al. [47] using the same IPAQ in a sample from the University of Vigo (Spain). Moreover, the results obtained here in the Spanish and Portuguese university students’ sample were better than those reported by Haase et al. [48], who focused on several countries worldwide, in which 46% of Mediterranean university women lead a sedentary lifestyle. Interestingly, the current results are in line with results reported in university women from North-Western Europe and the United States (24% of inactive students) [48]. The study of the profile of physical activity in relation to the consumption of cocoa provided an inverse correlation between cocoa consumption and physical activity, in such a way that the more cocoa they ate, the less exercise they performed. This fact could also be seen when studying the proportion of people with low and high physical activity in the groups of students with low and high consumption of cocoa. This analysis shows that in the higher consumer group, the percentage of students with high activity was lower than that in the lower consumer group, and the proportion of students with low activity was higher than that in the group consuming less cocoa. These results could be partially explained according to Drenowatz et al. [49], who reported the relation between hedonic appetite sensations and exercise in the young population. Drenowatz et al. found that walking was associated with craving for chocolate, whereas aerobic exercise was associated with craving for fruits [49]. In addition, these results could also be attributed to the fact that sedentary students may have more free time than active students who have less time to spare eating chocolate. A third possibility would be that the high activity students tend to have a healthier lifestyle, including less chocolate consumption. These hypotheses remain to be clarified in further studies involving athletes.

Regarding the health questionnaire, the university students reported, logically, a good health status, far away from suffering chronic diseases involving neoplasm and cardiovascular diseases, the main causes of death in the Spanish population [37]. The most frequently reported chronic conditions by university students in the health questionnaire were headaches and migraines. In general, the moderate cocoa consumption group has significantly less chronic disease prevalence than low consumers. Although further studies with an older population and bigger sample must be performed, the recommendation for moderate cocoa consumption can be reinforced. In line with this, there is some controversy due to the possible role of chocolate in migraine and headache [50]. On the one hand, a high chocolate consumption has been suggested as a food trigger for migraines and headaches [51], in spite of the fact that no biological mechanisms have been clearly identified to date and there is no solid scientific evidence that this is the case [52]. However, on the other hand, there is also a hypothesis suggesting that migraines may derive from IgE and IgG-mediated food hypersensitivity, which could be ameliorated by cocoa polyphenols with immunomodulatory effects [51].

With regard to other indicators of disease, no pattern related to cocoa consumption has been found, for example in the case of flu, diarrhoea or some others. This is also the case for the referred mean BP. No effect of cocoa consumption has been found in this study, although the cocoa’s effect on lowering BP is widely described [53] and there is a claim from the European Food Safety Authority (EFSA) correlating cocoa consumption and cardiovascular benefits such as producing vasodilatation [54]. This can be explained by the fact that studies demonstrating the anti-hypertensive effect of cocoa were, in general, carried out in subjects with high BP [28]; however, in our case, most of the students had a BP within the normal range. Furthermore, it must be considered that the EFSA claim recommends the consumption of 200 mg cocoa flavanols per day [54], which is about three times the average consumed by the participants in the current study (13 g cocoa/day, which provides 66 mg of cocoa flavanols).

The possible relationship between cocoa consumption and allergy prevention is supported by the evidence of flavonoid influence on the immune system, particularly on the T-helper 1 and T-helper 2 balance (Th1/Th2) [55], on allergy and asthma [56] and on our previous studies developed with cocoa-enriched diets in several animal models of allergy [26,57,58,59]. Regarding flavonoid effects on allergy, preclinical studies, mainly carried out in rodents, suggest that they may have a role in the prevention of IgE synthesis and mast cell degranulation [56]. After considering the results from animal models with allergic asthma, it has been suggested that preventive treatment with particular flavonoid classes reduced airway hyper-responsiveness, which was accompanied by reduced inflammatory mediator release, such as histamine and cytokines, as well as by lower cell infiltration [56]. In short, some particular flavonoids could be used as an alternative or complementary therapy in the prevention and treatment of some allergies. Nevertheless, an increased number of clinical trials are required in order to confirm the therapeutic role of flavonoids. The relationship between cocoa consumption and allergies was studied here, considering the three student groups according to their cocoa intake and self-reported allergy disease. Interestingly, the percentage of allergic people in those people consuming more than 7 g/day (moderate and high consumer groups) was lower than that in the low consumer group. Moreover, the moderate cocoa intake was associated with a lower presence of symptoms. All these results enable us to suggest that regular cocoa consumption could be related to the prevention or amelioration of the health imbalance induced by allergic processes. This relationship is supported by in vitro and preclinical effects of cocoa flavonoids on several allergic mechanisms, such as reducing mediators’ release [55], shaping the Th1/Th2 response, and down-modulating the IgE production [26]. The results obtained here are in line with those from a large multinational cross-sectional study in European adults (GA^2^LEN), in which an association between total flavonoid intake—and in particular pro-anthocyanidins—and ventilatory function and spirometric restriction, in a positive and negative way, respectively, was observed [60].

Despite the robust associations found in the current study, some limitations can be observed. The number of participants should be increased to confirm the hypothesis concerning the anti-allergic effects of cocoa. In addition, besides cocoa, the diet of the participants—and the content of flavonoids in this diet—may also have a role in these effects. For that reason, an ongoing study will aim to evaluate the impact of both an overall and particular type of flavonoid intake on allergies. Finally, some dietary habits can be linked to a healthy lifestyle in general, and although we considered lifestyle variables, unmeasured confounding factors cannot be ruled out. These limitations must encourage further studies, which should include a larger number of participants and a wider range of ages.

## 4. Materials and Methods

### 4.1. Participants

The present study was conducted according to the guidelines laid down in the Declaration of Helsinki and the study protocol was approved by the Ethical Committee of the University of Barcelona (IRB 00003099) and by the Egas Moniz Ethical Commission (ref. 331). Written informed consent was obtained from all participants after the aims and procedure of the study were explained by members of the research team. The study population was recruited among students from several Health Science graduation and postgraduation programmes in the Faculty of Pharmacy and Food Science at the University of Barcelona and in the Egas Moniz Health Sciences Institute of Portugal. The total sample included was 270 (199 females and 71 males), with a mean age of 22.55 ± 3.94 years. Data were collected in the school year 2013 to 2014.

The main sociodemographic characteristics of the sample are summarized in Table 5. Values of weight and height were considered normal: ~70 kg and 1.75 m for males and ~59 kg and 1.65 m for females. The mean BMI for all participants together was 21.9 ± 2.8 kg/m^2^, only 7% of the participants had a BMI lower than 18.5 kg/m^2^ and 11.5% had a BMI higher than 25 kg/m^2^. Around 70% of the students lived with their family, 28% with flatmates, and only 2.6% lived alone.

### 4.2. Procedure and Assessment

The participants were required to respond to a previously validated FFQ designed to assess chocolate and cocoa consumption by means of 90 items [27]. Briefly, the FFQ inquiries, among others, about the consumption of cereals, dairy products, spreads and confectionery (pastries and snacks) which can include chocolate. In addition, the kind of chocolate bars consumed by the participants (white, with milk or with <60%, 60–70%, 70–85%, >85% cocoa) was asked. After that, and taking into account the portions consumed, the amount of cocoa ingested by each participant was calculated. In addition, at the same time, they answered the IPAQ (https://sites.google.com/site/theipaq/home), which measures a population’s physical activity. Finally, students completed an ad hoc health status questionnaire, which included questions about chronic diseases and episodes of fever, flu, diarrhoea, or illnesses in general during the previous month. The questionnaires were answered during the period from May to October in 2014.

### 4.3. Cocoa Consumption Assessment

From the data obtained in the FFQ, the amount of cocoa intake was established in the 270 students as in previous studies [27]. According to the data, the sample of 270 individuals was divided into three groups of similar size and with different levels of cocoa consumption. The first group was made up of those individuals who ingested less than 7 g of cocoa daily (low consumers), the second group comprised those with a consumption of between 7 and 15 g (moderate consumers) and the third group consisted of individuals consuming more than 15 g per day (high consumers). The amount of cocoa in low consumers is equivalent to eating less than 5 g/day of 70%-cocoa chocolate, which means eating one chocolate portion (20 g) at a frequency of less than every 4 days. The amount of cocoa in moderate consumers ranged between 5 and 10 g of 70%-cocoa chocolate and represents eating one chocolate portion every 2–4 days. In the high consumer group, the frequency of eating one chocolate portion is higher than one every 2 days.

### 4.4. International Physical Activity Questionnaire (IPAQ)

Physical activity was assessed through the short version of the International Physical Activity Questionnaire (IPAQ), which has been used in other studies performed in young adults and in university students [47,61,62]. This questionnaire consists of four general questions, is available to use in a self-administered way and is suitable for national population prevalence studies on participation in physical activity. Participants were asked to think about the activities they do, such as working in the garden and in the house, getting from one place to another, and, in their spare time, exercise or sport. The data obtained from the IPAQ short form included two types of results. Firstly, categorical data that allow us to classify individuals within three categories of physical activity: inactive, minimally inactive, and health-enhancing physical activity (HEPA). Secondly, continuous indicators of physical activity presented as median minutes or median metabolic equivalents of task (METs) per minutes can be obtained. Median values can be computed for walking (W), moderate-intensity activities (M), and vigorous-intensity activities (V). The continuous score is expressed as METs-min/week for each activity (W, M or V), assessed from the METs level multiplied by minutes of activity multiplied by events per week.

### 4.5. Health Status Questionnaire

The participants answered a questionnaire providing information regarding their health status. The questionnaire, created ad hoc for this purpose, was partially based on that used in the ‘Enquesta nutricional de la població Catalana (ENCAT)’ 2003 [63]. In our case it had the following sections: (1) questions related to anthropometrical values including weight and height; (2) questions about lifestyle habits, the self-evaluation of health status (including BP and cholesterol values, if the student suffered from diabetes, anaemia, asthma, chronic bronchitis, varicose veins, eating disorders, migraine or frequent headaches, cataracts, chronic cervical, lumbar or dorsal back pain, arthrosis, arthritis, osteoporosis, prostate problems, urinary incontinence, chronic constipation, haemorrhoids, stomach or duodenal ulcer, chronic skin problems, thyroid problems, depression / anxiety, apoplexy, myocardial infarction, malignant tumours, among others), usual medication and nutritional supplements; and (3) questions about the immune function assessment. This last section, as one of the main objectives of the study, was expanded with respect to the corresponding ENCAT section, focusing on questions regarding not only the presence of allergies and intolerances but also the frequency of symptoms of this phenomenon, its treatment and resolution.

The mean of BP (mean arterial pressure) was calculated by adding the systolic blood pressure to the double of the diastolic blood pressure, and dividing by 3.

### 4.6. Statistical Analysis

Statistical analysis was performed by the software IBM Statistical Package for the Social Sciences (SPSS, version 22.0, Chicago, IL, USA). Levene’s test was carried out to assess the homogeneity of variance and the Shapiro−Wilk test to evaluate the distribution of the results. The conventional one-way analysis of variance (ANOVA) test followed by the Bonferroni post hoc test was performed when there was a normal distribution and equality of variance existed. On the other hand, the results having different variance and/or different distribution were evaluated by the non-parametric Kruskal−Wallis test, followed by the Mann−Whitney U post hoc test. Comparison between proportions was performed by the chi-squared test. Significant differences were established at *p* < 0.05.

## 5. Conclusions

After conducting a cross-sectional, observational, pilot study, we have found an inverse relationship between cocoa consumption in humans and allergy prevalence. This fact is of importance because of the relationship between polyphenol-rich foods and human health as well as the high amount of polyphenols in cocoa. Therefore, it can be suggested that a diet including a moderate consumption of cocoa products might substantially prevent allergic diseases and might ameliorate allergy symptoms. This study opens the door to further studies that could be performed with an increased number of participants, to assess the impact of cocoa consumption on this and other immune-mediated diseases and shed light on the immunoregulatory effect of cocoa in humans.

## Figures and Tables

**Table 1:** Gender and age distribution of low, moderate and high cocoa consumers. Results are expressed as percentage or mean ± standard deviation of the mean (SDM). Different letters mean statistical difference.

		Low Consumers (<7 g)	Moderate Consumers (7–15 g)	High Consumers (>15 g)
Cocoa intake (g)		4 ± 2 ^a^	11 ± 2 ^b^	25 ± 9 ^c^
*N*		87	91	92
Gender	Female (%)	74% ^a^	70% ^a^	77% ^a^
	Male (%)	26% ^a^	30% ^a^	23% ^a^
Age (y)		23 ± 3 ^a^	22 ± 4 ^a^	22 ± 4 ^a^

*N* is the number of individuals in each group.

**Table 2:** BMI, smoking habits and physical activity in low, moderate and high cocoa consumers. Results are expressed as mean ± SDM or ratio. Different letters mean statistical difference.

	Low Consumers	Moderate Consumers	High Consumers
BMI	22 ± 3 ^a^	22 ± 3 ^a^	22 ± 3 ^a^
Non-smokers	75% ^a^	68% ^a^	68% ^a^
Current smokers	21% ^a^	20% ^a^	22% ^a^
Ex-smokers	4% ^a^	12% ^b^	10% ^b^
METs/week	3002 ± 2735 ^a^	2516 ± 2280 ^a^	2573 ± 2580 ^a^

**Table 3:** Indicators of disease for the three cocoa consumption groups. Values are expressed as mean ± SDM or proportions in each subgroup (*N* = 87–92). Different letters mean statistical difference.

	Low Consumers	Moderate Consumers	High Consumers
Mean of blood pressure (mm Hg)	87 ± 9 ^a^	87 ± 8 ^a^	87 ± 9 ^a^
Hypertension (%)	2% ^a^	3% ^a^	1% ^a^
Chronic diseases (%)	64% ^a^	51% ^b^	59% ^ab^
Fever (%)	8% ^a^	5% ^a^	11% ^a^
Flu (%)	32% ^a^	32% ^a^	29% ^a^
Diarrhoea (%)	0% ^a^	1% ^a^	1% ^a^
Other illnesses (%)	9% ^a^	7% ^a^	10% ^a^

**Table 4:** Characteristics of allergy in allergic students of the three groups according to their cocoa intake. Results are expressed as percentage or mean ± SDM (*N* = 12–24). Different letters mean statistical difference.

	Low Consumers	Moderate Consumers	High Consumers
Frequency of allergic symptoms (days/month)	6 ± 10 ^a^	2 ± 2 ^a^	4 ± 6 ^a^
People with allergy once a month (%)	50% ^a^	28% ^b^	59% ^a^
Cutaneous symptoms (%)	42% ^a^	25% ^b^	35% ^ab^
Oropharynx symptoms (%)	38% ^a^	25% ^b^	35% ^ab^
Respiratory symptoms (%)	17% ^a^	0% ^b^	12% ^ab^
Digestive symptoms (%)	0% ^a^	0% ^a^	0% ^a^
Absenteeism due to allergy (days)	0.2 ± 0.7 ^a^	0.0 ± 0.0 ^a^	0.1 ± 0.3 ^a^
People with allergy treatment (%)	54% ^a^	33% ^b^	59% ^a^

**Table 5:** Sociodemographic characteristics of participants who completed the study. Values are expressed as mean ± SDM, as well as the range (in squared brackets), and the proportion for anthropometrical and demographic data.

	Undergraduate Students		Graduate Students		Total
	Men (*N* = 63)	Women (*N* = 140)	Men (*N* = 8)	Women (*N* = 59)	(*N* = 270)
Age (y)	21 ± 3	21 ± 3	25 ± 4	26 ± 3	22 ± 4
	[19–32]	[18–41]	[23–31]	[41–90]	[18–42]
Weight (kg)	71 ± 11	59 ± 9	70 ± 11	60 ± 3	62 ± 11
	[43–103]	[35–100]	[48–89]	[41–90]	[41–100]
Height (m)	1.7 ± 0.1	1.6 ± 0.1	1.7 ± 0.1	1.6 ± 0.0	1.7 ± 0.1
	[1.5–1.9]	[1.5–1.9]	[1.5–1.9]	[1.5–1.9]	[1.5–1.9]
BMI (kg/m^2^)	23 ± 3	21 ± 3	23 ± 2	22 ± 3	22 ± 3
	[16–29]	[16–33]	[21–29]	[16–28]	[16–33]
**Residential status, %**					
Alone	2%	1%	22%	5%	3%
With family	84%	63%	44%	37%	61%
Own family	5%	5%	0%	20%	8%
Flatmates	10%	31%	33%	37%	28%
